# Experimental Study on the Application of Polymer Agents in Offshore Oil Fields: Optimization Design for Enhanced Oil Recovery

**DOI:** 10.3390/polym17020244

**Published:** 2025-01-20

**Authors:** Xianjie Li, Jian Zhang, Yaqian Zhang, Cuo Guan, Zheyu Liu, Ke Hu, Ruokun Xian, Yiqiang Li

**Affiliations:** 1 State Key Laboratory of Offshore Oil and Gas Exploitation, Beijing 100028, China; lixj8@cnooc.com.cn (X.L.); zhangjian@cnooc.com.cn (J.Z.); guancuo@cnooc.com.cn (C.G.); huke@cnooc.com.cn (K.H.); xianrk@cnooc.com.cn (R.X.); 2 CNOOC Research Institute Co., Ltd., Beijing 100028, China; 3 State Key Laboratory of Petroleum Resources and Engineering, China University of Petroleum (Beijing), Beijing 102249, China; yaqianz_cup@163.com (Y.Z.); yiqiangli@cup.edu.cn (Y.L.); 4 College of Petroleum Engineering, China University of Petroleum (Beijing), Beijing 102249, China

**Keywords:** strong heterogeneity, discontinuous chemical flooding, polymer slug combination, displacement equilibrium

## Abstract

The Bohai oilfield is characterized by severe heterogeneity and high average permeability, leading to a low water flooding recovery efficiency. Polymer flooding only works for a certain heterogeneous reservoir. Therefore, supplementary technologies for further enlarging the swept volume are still necessary. Based on the concept of discontinuous chemical flooding with multi slugs, three chemical systems, which were polymer gel (PG), hydrophobically associating polymer (polymer A), and conventional polymer (polymer B), were selected as the profile control and displacing agents. The optimization design of the discontinuous chemical flooding was investigated by core flooding experiments and displacement equilibrium degree calculation. The gel, polymer A, and polymer B were classified into three levels based on their profile control performance. The degree of displacement equilibrium was defined by considering the sweep conditions and oil displacement efficiency of each layer. The effectiveness of displacement equilibrium degree was validated through a three-core parallel displacement experiment. Additionally, the parallel core displacement experiment optimized the slug size, combination method, and shift timing of chemicals. Finally, a five-core parallel displacement experiment verified the enhanced oil recovery (EOR) performance of discontinuous chemical flooding. The results show that the displacement equilibrium curve exhibited a stepwise change. The efficiency of discontinuous chemical flooding became more significant with the number of layers increasing and heterogeneity intensifying. Under the combination of permeability of 5000/2000/500 mD, the optimal chemical dosage for the chemical discontinuous flooding was a 0.7 pore volume (PV). The optimal combination pattern was the alternation injection in the form of “medium-strong-weak-strong-weak”, achieving a displacement equilibrium degree of 82.3%. The optimal shift timing of chemicals occurred at a water cut of 70%, yielding a displacement equilibrium degree of 87.7%. The five-core parallel displacement experiment demonstrated that discontinuous chemical flooding could get a higher incremental oil recovery of 24.5% compared to continuous chemical flooding, which presented a significantly enhanced oil recovery potential.

## 1. Introduction

Polymers are widely applied in various fields such as construction, healthcare, and energy [1,2,3]. One of their most extensive applications is in enhancing oil recovery through chemical flooding processes [4]. Currently, polymers in common use are classified into two types: synthetic polymers and biopolymers [3,4,5]. Synthetic polymers are artificially synthesized by the polymerization of acrylamide monomers and are often modified based on the requirement of the reservoir [6,7]. Hydrolyzed polyacrylamide (HPAM) is the most common and widely used synthetic polymer both at the field and experimental level due to its cost-effective nature [6,8]. Most synthetic polymers are PAM derivatives [4]. Polymer flooding can significantly increase crude oil recovery factors and is widely used among existing chemical flooding techniques, with broad application prospects [9,10]. The mechanisms for enhancing recovery primarily involve increasing the viscosity of the aqueous phase and reducing the aqueous phase permeability, thereby improving the oil–water mobility ratio, enhancing the vertical water absorption profile and increasing the areal sweep efficiency [8,9,10]. Additionally, at the microscopic scale, due to the polymer’s (i) pulling effect, (ii) stripping effect, (iii) oil thread phenomenon, and (iv) shear thickening effect, polymers can mobilize residual oil trapped by capillary forces and rock structures, thus improving microscopic oil displacement efficiency [9,10,11]. As a mature chemical flooding technology for enhanced oil recovery, polymer flooding is widely applied in onshore oilfields such as Daqing, Dagang, Yumen, and Shengli, and has achieved remarkable results in improving oil recovery factors [8,9,12].

Offshore oilfields typically comprise loose sandstone with high permeability, strong reservoir heterogeneity, and high oil viscosity. The large well spacing, multilayer commingled development, and strong injection and production will result in the severe channeling of injected fluids, ultimately leading to a low water flooding efficiency. There are still a lot of residual oil remains after water flooding in the reservoirs [13,14,15]. Polymer flooding can further expand the sweep volume based on water flooding, and is currently an effective technique for enhancing oil recovery (EOR) in offshore oilfields. However, the late stage of polymer flooding is inevitably accompanied by profile reversal, reducing the reservoir control capacity and leaving approximately 50% of the more dispersed oil in the formation [7,16,17]. Numerous laboratory and field tests have shown that polymer flooding can only increase oil recovery by about 10–12% over water flooding [18,19,20,21]. Furthermore, in the high-water-cut stage of offshore oilfields, reservoir heterogeneity is further exacerbated, and the profile control ability of polymers is limited. In the presence of dominant flow channels and large pores, the polymer cannot prevent channeling. Therefore, supplementary technologies are needed to address the limitations of polymer flooding in expanding the swept volume in offshore oilfields [22,23,24].

Foam flooding, gel particle flooding, and multiphase thermal fluid flooding can effectively alleviate fluid channeling and further enhance oil recovery in offshore oilfields [25,26,27,28]. However, these methods are more complex than polymer flooding. Based on the concepts of multi-slug injection of polymers [29] and stepwise injection [30], Zhang et al. proposed the theory and model of discontinuous chemical flooding for high-water-cut offshore oilfields [31,32]. This technology designs different concentrations and molecular weights of polymers, hydrophobically associating polymers, gel particles, and gels as slug combinations according to reservoir conditions, effectively controlling injection pressure and significantly increasing the swept volume while plugging channeling paths. The technology has achieved good field application results, but it is still in the laboratory research stage. Two main issues need further clarification: (1) how to evaluate the effect of discontinuous chemical flooding. Displacement equilibrium is commonly used to evaluate the development degree of heterogeneous reservoirs [20,31,32], but current indicators are difficult to monitor and obtain during laboratory and field applications, requiring a more straightforward and accurate method to evaluate the development effect of discontinuous chemical flooding. (2) Optimization design of slug combinations for discontinuous chemical flooding. The total injection volume of the polymer solution, the injection volume of each agent, the combination method, and the conversion timing all play a key role in the effect of discontinuous chemical flooding and require further laboratory research to provide a basis for field parameter optimization.

The evaluation method and parameter optimization design of discontinuous chemical flooding were systematically researched. First, the equilibrium displacement degree was defined based on the number of layers in heterogeneous reservoirs and the oil recovery factor in individual layers. To verify its feasibility and sensitivity, discontinuous flooding and single polymer flooding scenarios were designed. Using a large flat-panel model, the optimal slug size for discontinuous chemical flooding was determined. Three different intensities of profile control systems were selected, and a three-core parallel displacement experiment was conducted to optimize the combination method and injection timing of the agents. Subsequently, a five-cores parallel displacement experiment was used to verify the beneficial effects of discontinuous chemical flooding. The established evaluation method for discontinuous chemical flooding, applicable to typical offshore oilfields, effectively solve the problem of further enhancing oil recovery in the mid/late stages of polymer flooding in high-water-cut offshore oilfields.

## 2. Experimental Equipment and Process

### 2.1. Experimental Materials

**Figure 1:**
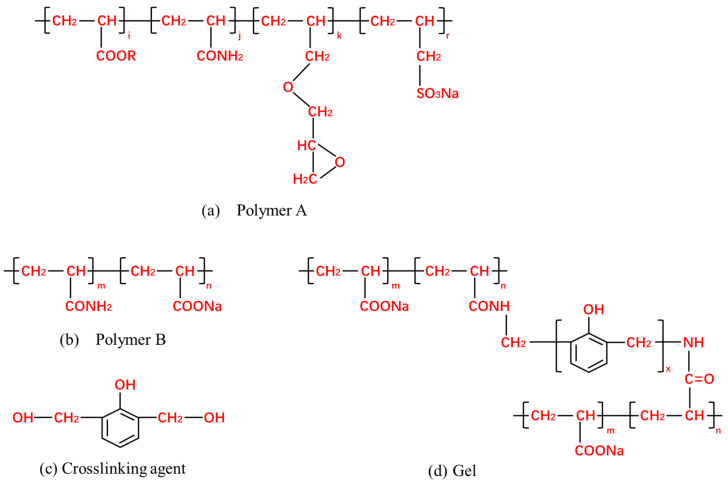
Molecular structure.

The polymer displacement agents included gel, polymer A, and polymer B. The relative molecular masses of polymers A and B were 3 × 10^7^ and 1 × 10^7^, respectively, and both are odorless, white, solid powders. The gel was formulated from polymer B and crosslinking agents in a specific ratio, and its molecular structure is illustrated in Figure 1. The experimental cores included a square gel-encapsulated core, 4.5 cm × 4.5 cm × 30 cm, with gas permeability values of 500 mD, 2000 mD, 5000 mD, 7500 mD, and 10,000 mD (the last two permeabilities were used in a five-core parallel displacement experiment). Porosity was approximately 30% and oil saturation was around 65%. A flat gel-encapsulated core, 60 cm × 60 cm × 5 cm, with gas permeability values of 500 mD, 2000 mD, and 5000 mD was also used.

The oil used in the experiment was a simulated oil composed of field crude oil blended with aviation kerosene, with a viscosity of 45 mPa·s at 60 °C. The water used was simulated formation water with a salinity of 7153.35 mg/L, and the detailed ion composition is shown in Table 1.

### 2.2. Experimental Equipment

Polymer agent performance evaluation: a MARS III high-temperature and high-pressure rheometer (temperature range 10–300 °C) (HAAKE Company, Vreden, Germany), a TC-150SD viscometer (Brookfield Company, Mumbai, Maharashtra), an electric stirrer (Waring Company, Vicksburg, MI, USA).

Core oil displacement experiment: the polymer displacement system consisted of an injection system, a core model, a liquid measurement system, a pressure control system, and a data acquisition system. Major equipment included an ISCO constant speed pump (ISCO Company, Louisville, KY, USA), a thermostatic chamber (up to 100 °C) (Hai’an Petroleum Research Instrument Company, Haian, China), pressure sensors and a data acquisition box (Hai’an Petroleum Research Instrument Company).

### 2.3. Experimental Methods

#### 2.3.1. Rheological Test

The rheological properties of the gel were tested using the HAAKE MARS III high-temperature and high-pressure rheometer. A prepared 12 mL sample was placed into a clean cylinder for stress and frequency scanning to obtain viscoelastic parameters.

#### 2.3.2. Viscosity Test

The viscosity of the polymer displacement agents at 60 °C was measured using a TC-150SD Brookfield viscometer. For the gel agents, rotor No. 3 was used (test range 0–20,000 mPa·s) and, for polymer A and polymer B systems, rotor No. 1 was used (test range 0–200 mPa·s). Gel solutions were prepared by mixing the polymer and crosslinking agents, and the resultant mix was then aged at 60 °C for 72 h. The viscosity of the system was tested every 6 h to obtain a viscosity curve as a function of aging time. A 12 mL sample was placed in the clean, dry cylinder, and the rotor speed was set to 6 r/min. After stabilization, viscosity data were recorded.

#### 2.3.3. Gel Strength Evaluation and Formula Optimization

First, the visual code method (Table 2) was used to select polymer concentrations that resulted in a gel strength between C and D and a suitable gelation time. Then, the optimal polymer-to-crosslinker ratio was determined using rheological parameters and viscosity variation methods [33].

#### 2.3.4. Definition of Displacement Equilibrium

The dynamic control degree of polymer flooding refers to the percentage of the pore volume in the oil layer that the polymer solution can affect under effective flow conditions, relative to the total pore volume of the oil layer. It reflects the extent of the reservoir sweep, which is related to reservoir pressure and dynamic resistance. The dynamic control degree of polymer flooding *E* is defined as:

(1) $$E=\frac{\sum_{i=1}^{m}a_{i}b_{i}V{\left(P\right)}_{i}}{\sum_{j=1}^{n}V_{i}}\times 100\%$$

(2) $$V(P)=\sum [\sum (S_{ji}(P)\cdot H_{ji}(P)\cdot \varphi )]\;j=1,i=1$$

where *V* is the pore volume of the oil layer that can be swept by the polymer solution and it is related to the effective driving pressure of the polymer. The terms *a_i_* and *b_i_* are dynamic control coefficients, related to dynamic resistance during polymer flooding. *S_ji_* represents the sweep area of the polymer flood network for the *i*-th well group in the *j*-th oil layer, *V_i_* is the total pore volume, *H_ji_* is the connection thickness of the injection–production well group that the polymer molecules can reach, and *ϕ* is the porosity.

Increasing the dynamic control degree of the polymer reservoir can achieve a more uniform sweep. However, since the parameters of the control degree are difficult to determine directly, a simplified parameter based on experiments is needed to represent the sweep balance, known as the displacement equilibrium. The function of displacement equilibrium is to describe the sweep effect of the displacement system. Theoretically, the greater the sweep efficiency, the larger the sweep range and recovery factor. Displacement equilibrium *λ* is defined as:

(3) $$\lambda =\xi \times \theta =\frac{n}{m}\times \frac{\eta_{\mathrm{ave}}}{\eta_{\max}}\times 100\%$$

where *λ* is the displacement equilibrium (as a percentage), *ξ* is the control coefficient, representing the ratio of the number of mobilized sub-layers *n* to the total number of sub-layers *m*, and *θ* is the homogeneity coefficient of mobilization, representing the ratio of the comprehensive recovery degree of each layer to the highest recovery degree of the layers.

#### 2.3.5. Optimization Design of Discontinuous Chemical Flooding

Based on the characteristics of the reservoir’s physical properties and thickness, artificial sandstone cores were designed according to the principle of similar permeability and thickness ratio, and displacement experiments were conducted. The experimental procedure was as follows:

1. Apply a vacuum of −0.1 MPa using a vacuum pump for 2 h.
2. Saturate the core with simulated water through self-absorption for 4 h, obtaining the pore volume *V_P_*.
3. Perform oil displacement with water using an ISCO pump at a speed of 0.5 mL/min. Once oil appears at the outlet, increase the flow rates to 1 mL/min and 2 mL/min in sequence. The total injected oil volume is twice the pore volume, and the initial oil saturation *S_oi_* is calculated.
4. Age the core for 3 days.
5. Connect the devices, fill the piston container with water and the polymer agent system, purge air from the pipelines and valves, and perform a pressure leak test at 2 MPa.
6. Then, proceed with the displacement experiment according to the experimental plan. Conduct water flooding. Upon reaching the predetermined water cut level, inject a slug of the designed chemical agent. Finally, conduct the subsequent water flooding until the composite water cut reaches 98%, at which point the experiment is terminated.
7. The process is conducted at the experimental temperature, monitoring pressure, and liquid production during displacement.
8. Data processing: calculate parameters such as fractional flow, oil recovery factor, and the equilibrium displacement degree using the following formulas.
  Fractional flow:
  (4) $$f_{w}=Q_{i}/\sum_{1}^{n}Q_{i}\times 100\%$$
  Oil recovery factor:
  (5) $$R=\sum_{1}^{n}V_{oi}/\left(V_{p}\times S_{oi}\right)\times 100\%$$
  Equilibrium displacement degree was calculated using Equation (3), where $Q_{i}$ represents the instantaneous liquid production of each layer, mL; *i* denotes the ith layer; and *n* is the total number of layers; $V_{oi}$ is the cumulative oil production of each layer, mL; $V_{p}$ is the pore volume, mL; and $S_{oi}$ is the oil saturation, %.

(1) Validation of Sweep Efficiency

**Figure 2:**
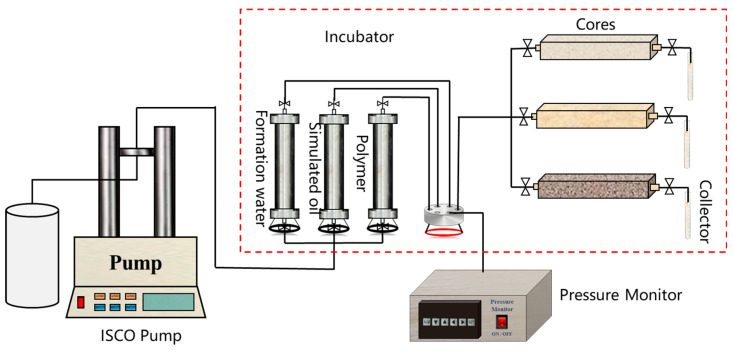
Experimental flow chart of the heterogeneous core model.

The sweep efficiency is defined in Section 2.3.4. Here, two sets of three-tube parallel core displacement experiments are designed. The displacement flow chart are shown in Figure 2. By comparing the recovery factors and sweep efficiency indicators in two-stage discontinuous chemical flooding and single polymer slug flooding, the feasibility of the sweep efficiency definition and its sensitivity to the experimental scheme were determined. The specific experimental plan is shown in Table 3.

(2) Design of Slug Size

**Figure 3:**
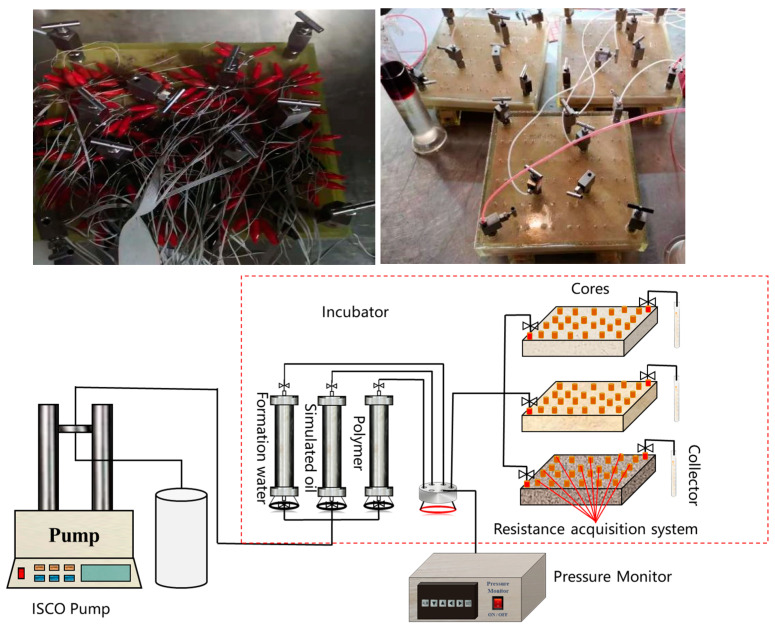
Physical diagram and flow diagram of the three-core large parallel model with electrodes.

The total size of the discontinuous chemical flooding slug was optimized using a three-plate parallel model. Electrodes and pressure points were arranged in the model. The physical model and displacement flow chart are shown in Figure 3. By injecting a large slug of the polymer system, the residual oil distribution characteristics were clarified using saturation electrodes, optimizing the best slug size. The specific experimental plan is shown in Table 4.

(3) Optimization of Combination Methods.

Based on the optimized conformance control system intensity, three discontinuous displacement combination methods were designed, including three-stage combinations, namely ① 0.2 PV gel + 0.2 PV polymer A + 0.3 PV polymer (strong–medium–weak), ② 0.3 PV polymer + 0.2 PV polymer A + 0.3 PV gel (weak–medium–strong), and ③ 0.2 PV polymer A + 0.2 PV gel + 0.3 PV polymer (medium–strong–weak), and a four-stage combination, namely ④ 0.2 PV polymer A + 0.1 PV gel + 0.15 PV polymer + 0.1 PV gel + 0.15 PV polymer (medium–strong–weak–strong–weak). These methods were evaluated through three-tube parallel displacement experiments (Figure 2) combined with sweep efficiency indicators, and the optimal system combination method was selected. The specific experimental plan is shown in Table 5.

(4) Optimization of the Best Conversion Timing

Based on the optimal system combination method, the conversion timing of each discontinuous chemical flooding system was studied. Similarly, using the three-tube parallel displacement experiment (Figure 2) combined with sweep efficiency indicators, the effect of improving reservoir heterogeneity at four different conversion timings was evaluated, determining the best combination method. The specific experimental plan is shown in Table 6.

#### 2.3.6. Verification of the Effectiveness of Discontinuous Chemical Flooding

To verify the beneficial effects of the discontinuous chemical flooding method optimized in Section 2.3.5, a five-tube parallel displacement experiment was conducted, comparing the discontinuous chemical flooding with continuous polymer flooding. The experimental steps followed those described in Section 2.3.5, and the experimental plan is outlined in Table 7.

## 3. Results and Discussion

### 3.1. Performance Evaluation of the Oil Displacement Systems

#### 3.1.1. Optimization of Gel System Formulation

The formulation design for gel application mainly included the design of polymer concentration and polymer-to-crosslinker ratio. Gel solutions were prepared using polymer concentrations of 1000, 1200, 1400, 1600, and 1750 mg/L, with a polymer-to-crosslinker ratio of 2:1. These solutions were then aged at 60 °C for a specified duration. The initial gelation time was defined as the intersection of the rapidly rising viscosity trend line with the x-axis. The stable gelation time was defined as the intersection of the trend line, where viscosity tended to stabilize with the rapidly rising trend line.

The results reveal that the solution with a concentration of 1750 mg/L underwent discoloration to yellow over time and exhibited a high gel strength. The results show that no good gel formation occurred at concentrations below 1600 mg/L, while, at 1750 mg/L, the gel strength was high, making it suitable for underground crosslinking to block high-permeability layers. Therefore, the optimal polymer concentration for the gel was between 1600–1750 mg/L.

**Figure 4:**
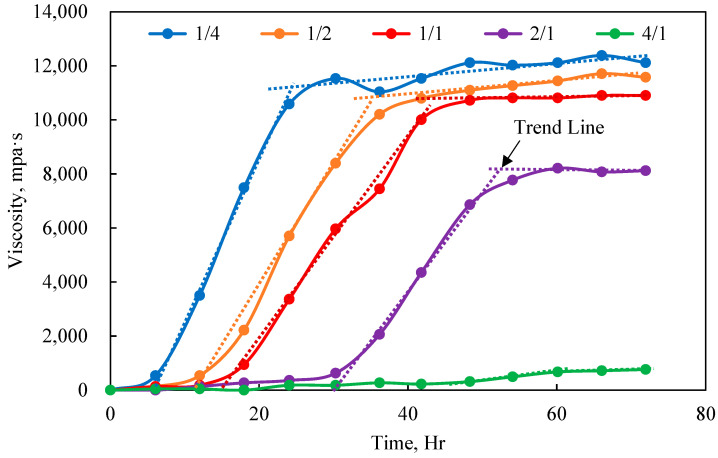
Viscosity curves of different polymer-to-crosslinker ratio systems.

A polymer with a concentration of 1600 mg/L was mixed with crosslinking agents at mass ratios of 4:1, 2:1, 1:1, 1:2, and 1:4 to obtain gel solutions. The viscosity curves were tested at 60 °C, and the experimental results are shown in Figure 4.

Figure 4 shows that the ratio of polymer to crosslinking agent significantly affected gel formation. A decreased polymer-to-crosslinker ratio, indicating an increased amount of crosslinking agent, enhanced the collision probability between the polymer and the crosslinking agent, leading to a reduced gelation time. Additionally, the quantity of gel formed increased, causing gel properties to progressively dominate the system and highlighting the difference in viscosity before and after gelation. Conversely, a high polymer-to-crosslinker ratio signifies a lesser amount of crosslinking agent, insufficient for complete crosslinking of the polymers in solution. This leads to partial crosslinking and the formation of a relatively small amount of gel. Consequently, the system primarily exhibited polymeric properties, resulting in a lower viscosity and an extended crosslinking time. Hence, the viscosity change in the system before and after gelation was insignificant. A polymer-to-crosslinker ratio of 1:1 or 1:2 yielded a suitable gelation time and gel strength, making this range the preferred one. Gel formation commenced after 12 h of aging, with a gelation rate of approximately 300 mPa·s/h, and the final gel strength could reach 10,000 mPa·s.

**Figure 5:**
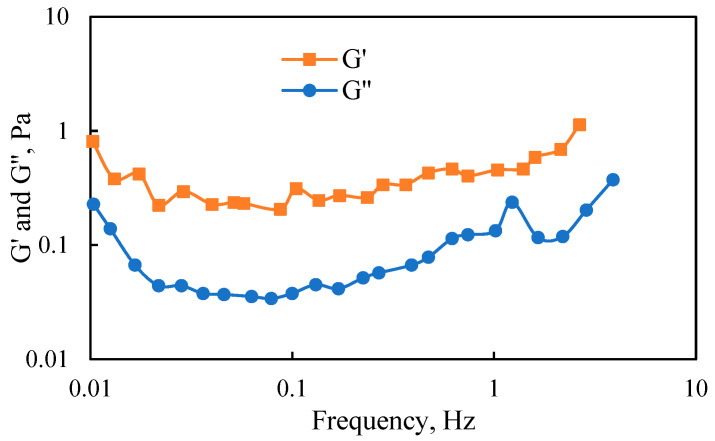
Relationship between viscoelastic moduli and vibration frequency of the gel under the optimal formula.

The viscoelasticity of the gel with a concentration of 1600 mg/L and a polymer-to-crosslinker ratio of 1:2 was tested, and the experimental results are shown in Figure 5. The results indicate that, after gel formation, across various vibration frequencies, the elastic modulus (G′) consistently exceeded the viscous modulus (G″), indicating a predominantly elastic system. Specifically, it was found that the elastic modulus G′ was less than 1 Pa. According to industry standards, this gel was classified as a weak gel.

#### 3.1.2. Graded Evaluation of the Performance of Oil Displacement Systems

The viscosity of polymer A and polymer B solutions at different concentrations was measured to determine their thickening properties, as shown in Table 8. The results indicate that the viscosity of the gel, as well as that of the polymer A and polymer B solutions, exhibited a stepwise variation. The viscosity of the gel was much higher than that of the polymer solutions, while the thickening ability of polymer A was stronger than that of regular polymers. As a result, the plugging strength of the three systems was classified into strong, medium, and weak levels.

### 3.2. Feasibility Verification of Displacement Equilibrium Evaluation Index

**Figure 6:**
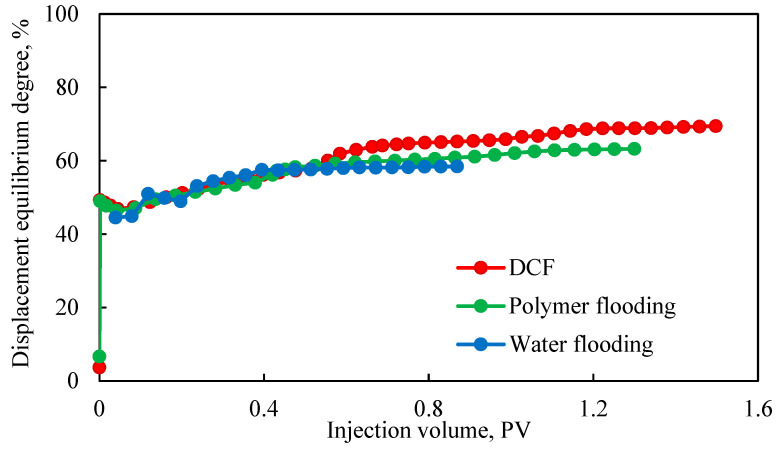
Comparison of discontinuous displacement and continuous flooding using displacement equilibrium degree.

Figure 6 shows the displacement equilibrium curves for two-stage discontinuous displacement and continuous displacement. The overall curves exhibit a stepwise change: initially, only the high-permeability layers were activated, resulting in a control coefficient of 1/3, which caused the first point to be significantly lower. As displacement progressed, the medium- and low-permeability layers were subsequently activated, with a control coefficient of 1, leading to a jump in the second point. Observing Figure 6, the equilibrium displacement degree did not show a significant increase in the water flooding scheme, yielding the smallest final equilibrium displacement degree and, consequently, the smallest corresponding recovery degree. In contrast, in the polymer flooding scheme, the equilibrium displacement degree exhibited an increase at an injection volume of 0.4 PV. The increase was because the chemical flooding started to take effect. During the DCF process, the equilibrium displacement degree showed two distinct steps at injection volumes of 0.5 PV and 1.1 PV. The injection of the second slug in DCF resulted in the reuse of the reservoir.

Table 9 presents key parameters from the three displacement experiments, revealing that discontinuous displacement can increase recovery by more than 8% compared to continuous polymer flooding, demonstrating significant enhancement effects. When comparing the final recovery and displacement equilibrium among the three experiments, it becomes evident that the recovery factor showed a more noticeable numerical difference than the displacement equilibrium, making it easier to compare the pros and cons of different schemes.

However, the displacement equilibrium index defined showed the activation of each layer through stepwise changes. As the number of small layers increased and the reservoir heterogeneity worsened, this advantage became more pronounced, making it suitable for the combined development of multilayer thick oil reservoirs offshore. Although the recovery factor showed more significant differences, Table 8 also shows that the displacement equilibrium still had noticeable differences, though less obvious than the recovery factor, and it can still be used to compare different schemes. Therefore, the displacement equilibrium parameter proposed can be used for the subsequent optimization of discontinuous chemical flooding schemes.

### 3.3. Combination System Slug Size Design

**Figure 7:**
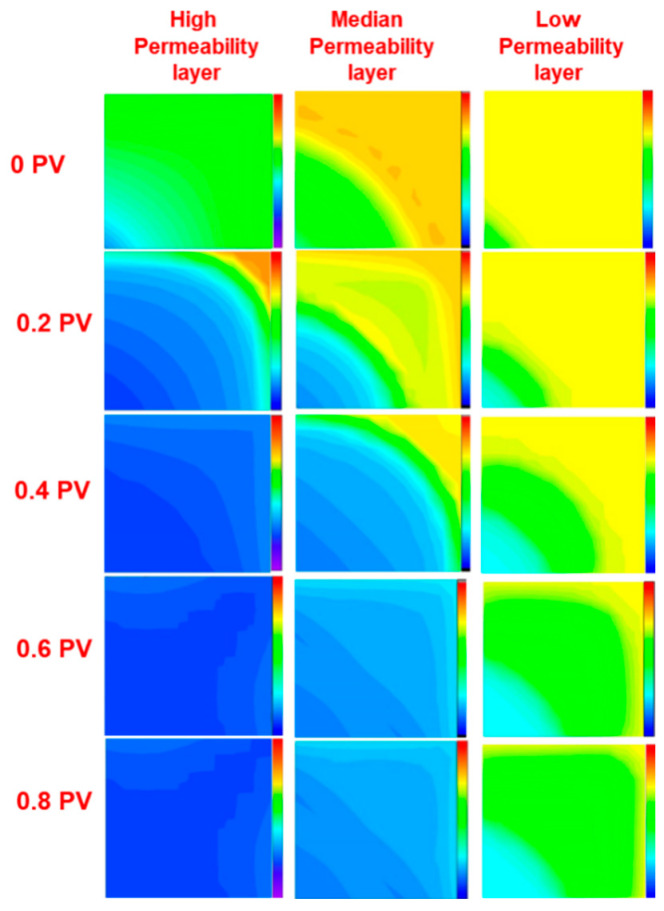
Oil saturation distribution diagram at different shift times of the combined system.

Figure 7 shows the oil saturation field maps at different injection pore volumes during the oil displacement experiment, as described in Table 4. At 0.2 PV, the oil saturation at the outlet end of the high-permeability layer was higher compared to that at 0.1 PV. In the medium-permeability layer at 0.2 PV, the oil saturation in the main streamline exhibited a trend of increasing, slightly decreasing, and then increasing again. This trend was attributed to the accumulation of crude oil during the chemical flooding process and its gradual displacement. After 0.4 PV, dominant channels gradually formed in the high-permeability layer. Consequently, the average residual oil saturation in the high, medium, and low-permeability layers at 0.6 PV showed minimal variation compared to that at 0.8 PV, with a variation of less than 5‰. This was due to the significant development potential and high displacement efficiency observed in the early stages of polymer solution injection. However, with continued injection, the advantage of high-permeability channels became more pronounced, greatly reducing the potential sweep volume. Therefore, there was little difference in the remaining oil saturation field after 0.6 PV.

Considering that the discontinuous chemical flooding extended the effective displacement period, a slug size of 0.7 PV was chosen as the optimal injection volume for subsequent investigations. This selection maximizes the recovery improvement while mitigating resource waste.

### 3.4. Optimization of the Combination Method for the Profile Control System

**Figure 8:**
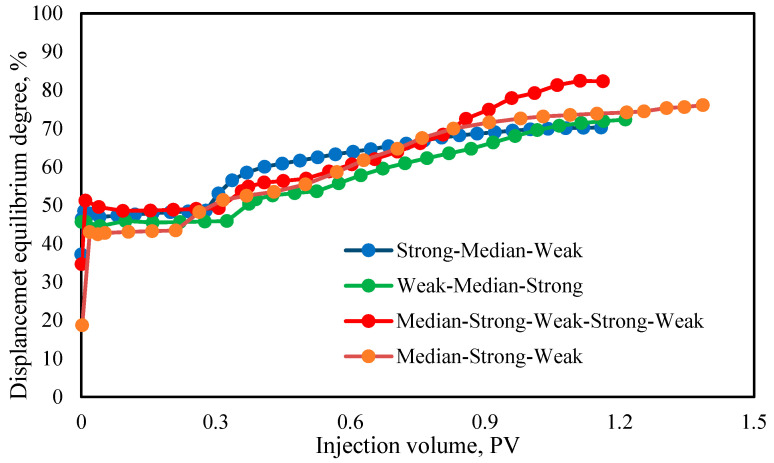
Displacement equilibrium degree curves for different discontinuous combination methods.

Figure 8 shows the displacement equilibrium degree curves under four different discontinuous chemical flooding combination methods. During the water flooding phase, the displacement equilibrium degree remained stable at around 50%. After the injection of the polymer solution, the displacement equilibrium degree rapidly increased and then stabilized again. Notably, each transition to a new polymer slug resulted in a discernible elevation in the displacement equilibrium degree.

For reservoirs with identical physical properties, they tended to have similar waterflooding development stages, including the affected formations and recovery degrees. Therefore, the displacement equilibrium degree during the waterflooding phase tended to be consistent among these reservoirs.

However, different chemical systems exhibited varying injectability in different formations, resulting in differential impacts on those formations when injected. Consequently, the displacement equilibrium degree exhibited varying trends.

For the strong–medium–weak combination, as the slug intensity decreased, the increase in the displacement equilibrium degree gradually weakened, due to the high-saturation oil wall formed by the strong slug during the initial stages of chemical flooding which hindered subsequent medium and weak slugs to re-coalesce the oil wall. Additionally, as production progressed, the remaining oil saturation gradually decreased, further complicating coalescence.

The displacement equilibrium degree for the medium–weak–strong combination exhibited two distinct steps. Initially, the chemical flooding coalesced the residual oil left by waterflooding, but, due to the weaker intensity of the chemical system, the growth rate was slower than that of the strong–medium–weak combination. Subsequently, the injection of the strong slug further enhanced the swept volume and coalesced the remaining oil. As production continued and the slug intensity decreased, the displacement equilibrium degree gradually stabilized.

Similarly, the weak–medium–strong combination also showed two distinct steps in the displacement equilibrium degree. Due to the different timing of slug injection, the results and growth rates of the displacement equilibrium degree differed from those of the medium–weak–strong combination.

Observing the medium–strong–weak–strong–weak combination, the displacement equilibrium degree exhibited three distinct steps. This characteristic was due to the secondary plugging of the high-permeability layer with a strong slug prior to breakthrough which facilitated the further development of the medium- and low-permeability layers, further increasing the displacement equilibrium degree.

By comparing the results of Figure 6 and Figure 8, the final displacement equilibrium degrees followed this order: five-stage > three-stage > two-stage discontinuous chemical flooding. Thus, the “medium-strong-weak-strong-weak” combination achieved the highest displacement equilibrium degree, indicating better displacement performance. The primary reason was that the five-stage displacement method advanced over the three-stage method by using smaller slug sizes of gel to effectively block high-permeability layers. This was subsequently followed by the utilization of polymer systems to efficiently displace oil from medium- and low-permeability layers. This approach avoids the problems of high pressure, low-permeability layer contamination, and long-term polymer channeling that could occur with large gel slugs.

The “strong-medium-weak” method resulted in the lowest displacement equilibrium degree, mainly because the gel’s blocking strength was too high, and injecting gel too early would hinder the full utilization of the remaining oil in the high-permeability layers.

The “medium-strong-weak-strong-weak” combination leveraged polymer A to fully displace high-permeability layers before using the gel to block them, allowing for the effective utilization of medium- and low-permeability layers. The alternation between strong and weak displacement helps avoid abnormal pressure increases caused by large gel slugs. Therefore, the optimal discontinuous chemical flooding combination was the “medium-strong-weak-strong-weak” configuration.

### 3.5. Optimization of the Best Switching Timing for the Profile Control System Combination

**Figure 9:**
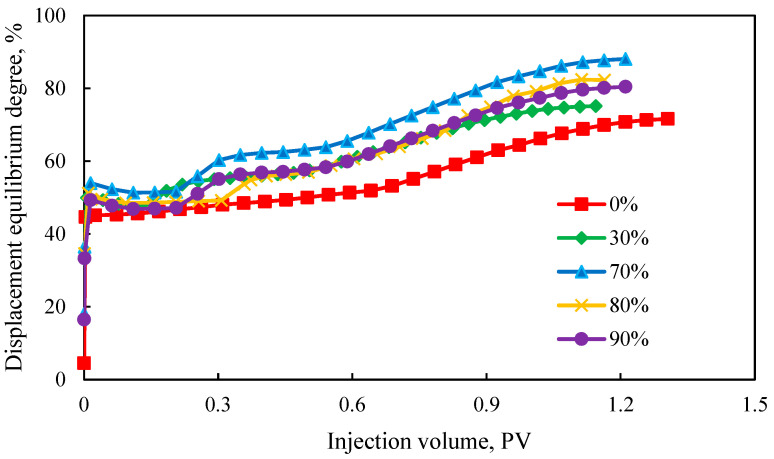
Displacement equilibrium degree curves at different injection times.

Figure 9 displays the displacement equilibrium degree curves under five distinct switching timings. The primary distinction among the various design schemes lied in the timing of chemical injection which resulted in varying residual oil saturation levels after waterflooding. However, the chemical systems and slug sizes remained consistent, leading to similar shapes in the displacement equilibrium degree curves. However, the rates of increase in the displacement equilibrium degree varied among different injection timings, due to the different distributions and saturation states of residual oil under different injection timings which affected the interaction and coalescence between the chemical agents and the crude oil. It can be observed that the highest displacement equilibrium degree, and, thus, the best displacement performance, occurred when the switching timing was at a 70% water cut. Conversely, the lowest displacement equilibrium degree, and the worst oil recovery factor, was observed when the switching occurred at a 0% water cut.

Switching to polymer flooding too early means that the full potential of water flooding is not exploited, resulting in a high oil saturation in the reservoir. This causes a rapid increase in injection pressure. Once channeling occurs in the high-permeability layers, mobilizing medium- and low-permeability layers becomes even more challenging. Additionally, switching too early significantly increases production costs. Conversely, switching too late results in a low oil saturation in the reservoir, with residual oil forming as oil films in small pores, making it difficult for the injected polymer solution to displace the oil.

Therefore, switching the polymer flooding system too early or too late is not beneficial for improving oil recovery. The optimal switching timing for discontinuous polymer flooding should be when the water cut is between 70% and 90%.

### 3.6. EOR Effect of Discontinuous Chemical Flooding

**Figure 10:**
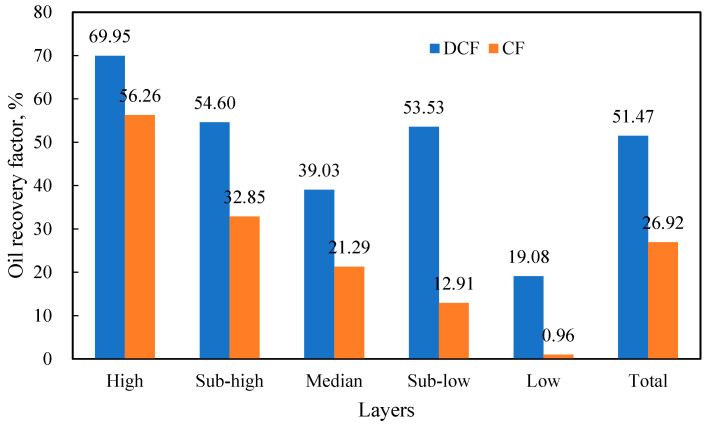
Comparison of recovery factors for different layers between DCF and CF in the five-core parallel flooding experiment.

Figure 10 compares the recovery factor of various layers in the five-tube parallel flooding experiments, utilizing discontinuous chemical flooding (DCF) and continuous chemical flooding (CF). The DCF achieved a final oil recovery factor of 51.47%, representing an increase of 24.55% compared to CF. This enhancement was primarily attributed to improved mobilization in the medium- and low-permeability layers. CF primarily mobilized high and secondary high-permeability layers, whereas DCF not only enhanced the recovery in high-permeability layers, but also significantly improved the mobilization in medium- and low-permeability layers. Notably, in DCF, the recovery factor of the medium-permeability layer was lower than that of the second-lowest permeability layer, due to gel contamination. This indicates that stronger gel plugging should only be applied after sufficient mobilization of the medium-permeability layers to prevent channeling.

**Figure 11:**
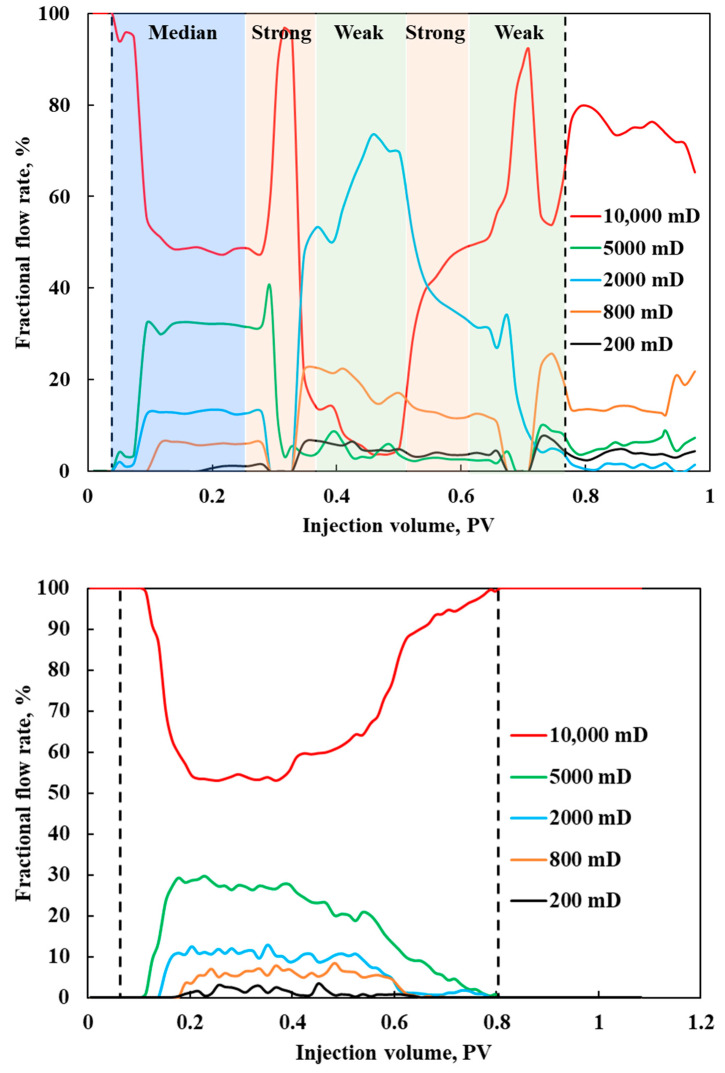
Comparison of fractional flow rate distribution between DCF and CF in five-core parallel flooding experiment.

Analyzing the above in conjunction with Figure 11, during the DCF process, the primary production phase for the sub-high permeability layer occurred during the initial slug injection. However, this initial strong slug effectively sealed off the sub-high permeability layer, resulting in suboptimal subsequent production. Conversely, the injection of two strong slugs blocked higher permeability layers, providing the sub-low permeability layer with two production opportunities. Consequently, the oil recovery of both layers is comparable.

Figure 11 compares the flow rate allocation between DCF and CF for each layer in the five-tube parallel flooding experiment. During the initial injection of polymer A, the flow rate in the high-permeability layer decreased to around 50%, enhancing mobilization in the medium- and low-permeability layers. However, continuing injection of a single slug system could revert the profile, with the high-permeability layer’s flow rate significantly increasing again. After the first gel slug injection in DCF, the flow rate in the medium-permeability layer improved significantly, but it subsequently fell below that of the second-lowest permeability layer. This observation supports the hypothesis of gel contamination in the medium-permeability layer. DCF effectively optimized the flow rate curves at each stage. Although no oil was ultimately recovered from any layer, the high-permeability layer’s flow rate remained below 80%, demonstrating its sustained effectiveness. The improvement of the injection and production profile in heterogeneous reservoirs is the key reason for the enhanced oil recovery achieved by DCF.

Upon injection of the chemical system in CF, the flow rate of the high-permeability layer decreased to approximately 50%. However, this effect was not sustained for a long period. The flow rate of the high-permeability layer significantly increased again at 0.4 PV, while the flow rates of the other layers remained below 15%. Based on this analysis, it is concluded that, in strongly heterogeneous multilayer reservoirs, the effectiveness of CF in improving the water injection profile is limited.

**Figure 12:**
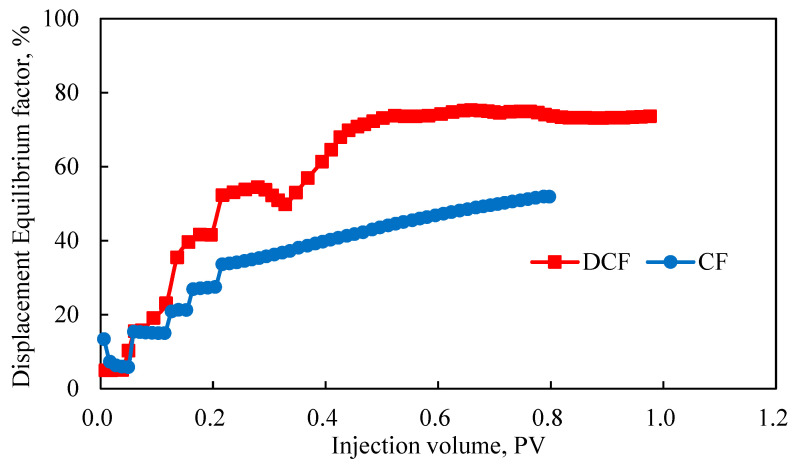
Comparison of displacement equilibrium degree between DCF and CF in five-core parallel flooding experiments.

Figure 12 presents a comparison of the displacement equilibrium degree for DCF and CF. The stair step pattern in the displacement equilibrium was more pronounced in the five-tube parallel flooding experiment. These curves distinctly showcase the activation of each layer. Initially, both DCF and CF exhibited similar displacement equilibrium degrees. However, during the continuous injection of polymer A, CF’s equilibrium degree increased slowly, whereas DCF’s equilibrium degree improved significantly. The observed decline in DCF’s displacement equilibrium stemmed from the widening gap between the overall recovery factor and the maximum recovery factor which is linked to the rapid mobilization of the high-permeability layer. The displacement equilibrium degree was influenced by two factors: the number of mobilized layers and the variations in mobilization among these layers. Throughout the CF process, the displacement equilibrium was clearly delineated into five stages, aligning with the data presented in Figure 11. These stages represent the sequential utilization of the five reservoir layers, leading to a gradual increase in the displacement equilibrium. As the displacement process progressed, the equilibrium exhibited a gradual increase before leveling off. This was mainly due to the oil recovery of the high-permeability layer, namely the maximum oil recovery, reaching over 50%, thereby constraining a further increase. Simultaneously, the growth rate of the comprehensive oil recovery also gradually decreased. These two factors interact, collectively shaping the final contour of the displacement equilibrium curve.

Ideally, all layers in DCF are mobilized (with the control coefficient of 1) and exhibit uniform recovery factors (with the homogeneity coefficient of 1), achieving a maximum equilibrium degree of 100%. Figure 12 affirms that displacement equilibrium serves as a valuable tool for assessing the efficacy of various displacement schemes and underscores the notable advantages of DCF.

## 4. Conclusions

The profile control and flooding performance of the gel, polymer A, and polymer B are evaluated based on parameters like viscosity and viscoelasticity, categorizing them into strong, medium, and weak grades. This study emphasizes the evaluation criteria for discontinuous chemical flooding (DCF) and the optimization of injection parameters through laboratory experiments. A displacement equilibrium degree is defined, considering the number and mobilization efficiency of mobilized layers. The slug size, combination strategy, and shift timing of DCF are optimized using the parallel core flooding experiments. The benefits of DCF in oil displacement are validated through five-core parallel flooding experiments. The specific conclusions are as follows:

1. The gel formula is optimized with a polymer concentration of 1600 mg/L and a polymer-to-gel ratio of 1:2. The gel exhibits strong viscoelasticity, with its elastic modulus significantly exceeding its viscous modulus. With a viscosity of 10,000 mPa·s, the gel is classified as weak. The profile control and flooding intensities of the three systems are rated as follows: gel—strong, polymer A—medium, and polymer B polymer—weak.

2. The displacement equilibrium degree reflects the mobilization efficiency and timing of each layer during DCF. The displacement equilibrium degree curve presents a stepwise pattern. The efficiency of DCF becomes more significant with the number of thin layers increasing and heterogeneity intensifying. This metric is highly sensitive to different flooding strategies, effectively highlighting differences among each displacement scheme.

3. Under the combination of a permeability of 5000/2000/500 mD, the optimal dosage for chemical discontinuous flooding is 0.7 PV. The optimal combination pattern is the injection in the sequence of “medium-strong-weak-strong-weak”. This pattern can achieve a displacement equilibrium degree of 82.3%. The optimal shift timing of polymers injection occurs at a water cut of 70%, yielding a displacement equilibrium degree of 87.7%.

4. In comparison to CF, DCF demonstrates a higher incremental oil recovery of 24.5% in the five-core parallel displacement experiment. DCF effectively blocks dominant flow channels and controls injection pressure, with its advantages being more significant in heterogeneous reservoirs.

## Figures and Tables

**Table 1:** Formation water ion composition.

Ion	Na^+^	K^+^	Mg^2+^	Ca^2+^	Cl^−^	SO_4_^2−^	Total
Concentration mg/L	2422.13	40.29	69.94	191.71	3971.52	17.67	7153.35
Ion	HCO_3_^−^	CO_3_^2−^	I^−^	Br^−^	B^−^		
Concentration mg/L	392.57	47.52	0.36	4.47	3.39		

**Table 2:** The gel strength code criteria [33].

Intensity Code	Gel Name	Corresponding Strength Description
A	Non-probabilistic gels	The system is no different from the polymer and is completely unbonded
B	High mobility gel	The viscosity of the system gradually increases and exceeds that of the polymer
C	Liquidity gel	Most of the gel can flow to the other end of the bottle
D	Medium flow gel	When flipping the glass bottle (<15% of the gel), the polymer cannot flow to the other end and often exists in the tongue type
E	Almost no flow of the gel	A small amount of the gel can flow slowly to the other end, and most of it is not liquid
F	High-shaped and immobile gel	The gel does not flow to the bottle mouth when turning around the glass bottle
G	Medium-shaped immobile gel	When flipped, it can only flow to the middle of the glass bottle
H	Slightly deformed immobile gel	Upon flipping, only the gel surface is deformed
I	Rigid gel	Upon flipping, the gel surface does not deform
J	Jolling gel	When shaking the glass bottle, you can feel the mechanical vibration like a sound fork

**Table 3:** Experimental scheme for the verification of displacement equilibrium degree.

No.	Core Permeability/mD	Transfer Timing	Experimental Scheme	Experimental Procedure
1	500/2000/5000	80%	The 0.7 PV polymer	Water flooding to a comprehensive water cut of 80%; polymer flooding of 0.7 PV after a water comprehensive water cut of 98%
2	500/2000/5000	80%	0.3 PV gel + 0.4 PV polymer	

**Table 4:** Experimental scheme of polymer slug size.

No.	Core Permeability/mD	Slug Size/PV	Experimental Scheme	Transfer Timing
1	500/2000/5000	1	Water flooding + 1 PV polymer + back water	Water cut of 80%

**Table 5:** Optimization design of oil displacement system combination methods.

No.	Core Permeability/mD	Slug Size	Combination Mode	Experimental Scheme	Transfer Timing
1	500/2000/5000	0.7	Strong–medium–weak	0.2 PV gel + 0.2 PV polymer A + 0.3 PV polymer + post-water	Water cut of 80%
2	500/2000/5000	0.7	Weak–medium–strong	0.3 PV polymer + 0.2 PV polymer A + 0.3 PV gel + post-water	Water cut of 80%
3	500/2000/5000	0.7	Medium–strong–weak	0.2 PV polymer A + 0.2 PV gel + 0.3 PV polymer + post-water	Water cut of 80%
4	500/2000/5000	0.7	Medium–strong–weak–strong–weak	0.2 PV polymer A + 0.1 PV gel + 0.15 PV polymer + 0.1 PV gel + 0.15 PV polymer + post-water	Water cut of 80%

**Table 6:** Optimization design of the shift timing for oil displacement experiments.

No.	Core Permeability/mD	Slug Size	Experimental Scheme	Transfer Timing
1	500/2000/5000	0.7	0.2 PV polymer A + 0.1 PV gel + 0.15 PV polymer + 0.1 PV gel + 0.15 PV polymer + back water (Medium–strong–weak–strong–weak)	Water cut of 0%
2	500/2000/5000	0.7		Water cut of 30%
3	500/2000/5000	0.7		Water cut of 70%
4	500/2000/5000	0.7		Water cut of 80%

**Table 7:** Five-core parallel displacement experiment schemes.

No.	Core Permeability/mD	Experimental Scheme	Experimental Procedure
1	500/2000/5000/7500/10,000	0.2 PV polymer A + 0.1 PV gel + 0.15 PV polymer + 0.1 PV gel + 0.15 PV polymer + post-water	Water flooding to a comprehensive water cut of 80%; polymer flooding post water to a total water cut of 100%
2	500/2000/5000/7500/10,000	The 0.7 PV polymer	

**Table 8:** Viscosity range of polymer agents.

No.	Polymer Agents	Main Control Parameters	Scope	Intensity
1	Gel	Dynamic viscosity	>2000	Strong
2	Polymer A	Dynamic viscosity	200–350 cP	Medium
3	Polymer B	Dynamic viscosity	30–50 cP	Weak

**Table 9:** Comparison of displacement equilibrium degree and oil recovery.

	Combined Recovery at a Water Cut of 80%, %	Ultimate Recovery, %	Final Displacement Equilibrium, %
Water drive	22.24	39.17	58.49
DCF	23.18	58.21	69.46
Polymer flooding	23.59	46.74	63.23

## Data Availability

The original contributions presented in this study are included in the article. Further inquiries can be directed to the corresponding author.
